# ﻿Two new species of *Squamosa* Bethune-Baker, 1908 (Lepidoptera, Limacodidae) and first female record of *S.chalcites* Orhant, 2000 from southern Asia

**DOI:** 10.3897/zookeys.1090.75823

**Published:** 2022-03-25

**Authors:** Jun Wu, Alexey V. Solovyev, Hui-Lin Han

**Affiliations:** 1 School of Forestry, Northeast Forestry University, Harbin, 150040, China; 2 Department of Biology and Chemistry, Ulyanovsk State Pedagogical University, Ulyanovsk, 432071, Russia; 3 Key Laboratory of Sustainable Forest Ecosystem Management, Ministry of Education, Northeast Forestry University, Harbin, 150040, China

**Keywords:** China, India, key, Myanmar, slug caterpillar moths, synonymy, taxonomy, Tibet

## Abstract

Two new species of the genus *Squamosa* Bethune-Baker, 1908 (Lepidoptera: Limacodidae): *S.medogensis***sp. nov.** and *S.undulophallus***sp. nov.**, are described from southern Asia. These new species are illustrated with images of the adults and male genitalia, and compared with similar species. A new synonymy is established for the subspecies *S.brevisuncabrevisunca* Wu & Fang, 2009 = *S.svetlanae* Solovyev & Witt, 2009, **syn. nov.** The female genitalia of *S.chalcites* Orhant, 2000 are illustrated and described for the first time. A distribution map for the new species and illustrations of Asian members of *Squamosa* are given, and a key to Asian species of the genus is also provided.

## ﻿Introduction

The genus *Squamosa* was erected by Bethune-Baker (1908), with *S.ferruginea* Bethune-Baker, 1908 as its type species [type locality New Guinea (Kebea)]. The second species of this genus, *S.ocellata* (Moore, 1879), was originally placed in the genus *Monema* until Hering (1931) transferred it to *Squamosa* in 1931. Thereafter, it was not until 2000 that a third species, *S.chalcites* Orhant, 2000, was described from Myanmar and Thailand by Orhant (2000). In 2009, an additional species, *S.brevisunca* Wu & Fang, 2009, was described, including two subspecies: *S.brevisuncabrevisunca* Wu & Fang, 2009 (=*S.svetlanae* Solovyev & Witt, 2009, syn. nov.) from China and Vietnam and *S.brevisuncayunnanensis* Wu & Fang, 2009 from Yunnan, China (Solovyev and Witt 2009; Wu and Fang 2009). Wu and Fang (2009) also clarified that the record of *S.ocellata* in China was based on a misidentification of *S.brevisunca*, and it had also been reported in Nepal and Bhutan (Cai 1981; Yoshimoto 1994; Irungbam et al. 2017). Later, Pan and Wu (2015) described a species, *S.monosa* Wu & Pan, 2015, from Xizang, China.

The moths belonging to this genus are of medium to large size. The antennae are broadly bipectinated at least in the basal half to three quarters, then serrate in the male and filiform in the female. The labial palpus is short, not quite reaching the vertex. The forewing has veins R_3-5_ stalked and R_2_ separated. The diagnostic external characters of the Asian *Squamosa* species are as follows: the forewing ground colour is yellow, with a conspicuous, large, rounded medial patch on the outside of the cell, and a narrow, curved, black subterminal line running from the costal margin to the tornus. The male genitalia have an apically bifid uncus and small gnathos; the valva is wide at the base and rounded at the cucullus; the saccular process is usually absent or in a well-developed hook-shape; the juxta is usually asymmetrical and rarely symmetrical; and the phallus is slender. The tibia spurs number 0–2–4. The type species of the genus, *S.ferruginea* Bethune-Baker, 1908, is known from New Guinea (Kebea). It differs considerably in appearance from the Asian members, and its male genitalia have not been described yet. As a consequence, clarification of the generic limits requires further investigation.

To date, the genus contains five described species ranging from New Guinea, India to China, including: *S.ferruginea* Bethune-Baker, 1908; *S.ocellata* (Moore, 1879); *S.chalcites* Orhant, 2000; *S.brevisuncabrevisunca* Wu & Fang, 2009; *S.brevisuncayunnanensis* Wu & Fang, 2009; and *S.monosa* Wu & Pan, 2015. Four species are described from Asia and three occur in China.

In this study, two species, *S.medogensis* sp. nov. and *S.undulophallus* sp. nov., collected from the southeast of Xizang Autonomous Region (= Tibet), China, as well as India and Myanmar, are described as new to science.

## ﻿Materials and methods

The specimens were collected with a 220V/450W mercury vapour lamp and a DC black light. Standard methods for dissection and preparation of the genitalia slides were used (Kononenko and Han 2007). The specimens were photographed using a Nikon D700 camera, whereas the genitalia slides were photographed with an Olympus photo microscope aided by the Helicon Focus software and further processed in Adobe Photoshop CS6.

The terminology of morphology follows Epstein (1996), and the following abbreviations are used in the figures:

**AA** apophysis anterioris;

**Aed** aedeagus;

**AP** apophysis posterioris;

**CB** corpus bursae;

**DB** ductus bursae;

**Gn** gnathos;

**Jx** juxta;

**PA** papillae anales;

**Sig** signa;

**SP** saccular process;

**Un** uncus;

**Va** valva.

All the type materials of the new species are deposited in the collection of Northeast Forestry University (**NEFU**), Harbin, China, except for five male paratypes of *Squamosaundulophallus* sp. nov., which are deposited in the Museum Witt München / Zoologische Staatssammlung München, Munich, Germany (**MWM/ZSM**). Material from the National Zoological Museum of China, Institute of Zoology, Chinese Academy of Sciences, Beijing, China (**IZCAS**) was also examined in this study.

## ﻿Taxonomic account

### ﻿Genus Squamosa

Taxon classificationAnimaliaLepidopteraLimacodidae

Bethune-Baker, 1908

994EC4E6-BC9B-5C86-8269-26A6ACCC2FEA

Squamosa Bethune-Baker, 1908, Novit. zool., 15: 183. Type species (original designation): Squamosaferruginea Bethune-Baker, 1908. Type locality New Guinea: Kebea.

### ﻿ Squamosa medogensis sp. nov.

Taxon classificationAnimaliaLepidopteraLimacodidae

B2C69A8E-880C-55DC-B577-BDEAC1A42EAF

http://zoobank.org/11275919-013D-46BC-A7EB-D30BC2D59A17

**Figures 1–12.**
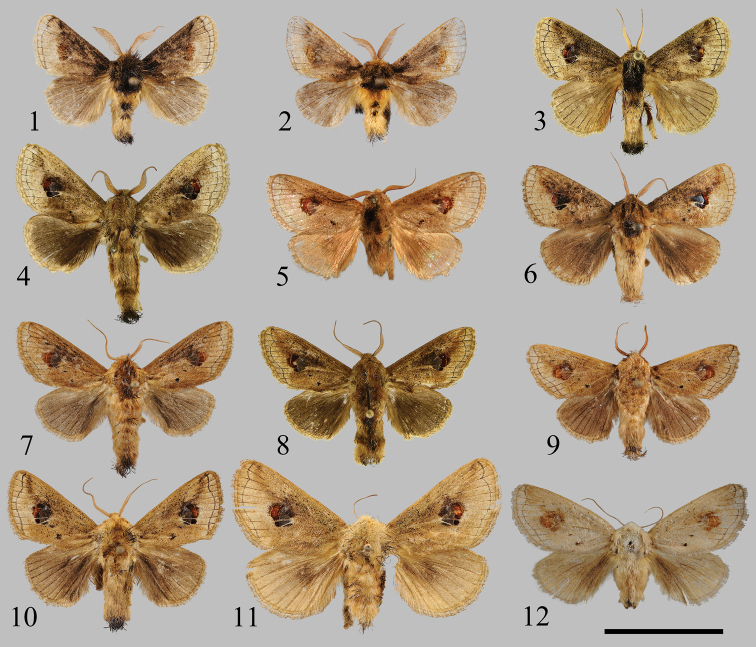
Adults of *Squamosa* spp.: **1, 2***S.medogensis* sp. nov., males, holotype (**1** in NEFU) and paratype (**2** in NEFU) **3***S.ocellata* (Moore, 1879), male, Sikkim, India (in MWM/ZSM) **4***S.brevisuncabrevisunca* Wu & Fang, 2009 (= *S.svetlanae* Solovyev & Witt, 2009, syn. nov., male, holotype) (in MWM/ZSM) **5***S.brevisuncabrevisunca* Wu & Fang, 2009, male, holotype (in IZCAS) **6***S.brevisuncayunnanensis* Wu & Fang, 2009, male, Yunnan, China (in NEFU) **7, 8***S.undulophallus* sp. nov., males, holotype (**7** in NEFU) and paratype (**8** in MWM/ZSM) **9***S.chalcites* Orhant, 2000, male, Chongqing, China (in NEFU) **10, 11***S.monosa* Wu & Pan, 2015, male, Xizang, China (**10** in NEFU) and female, Xizang, China (**11** in NEFU) **12***S.chalcites* Orhant, 2000, female, Chongqing, China (in NEFU). Scale bar: 2 cm.

**Figures 13–24.**
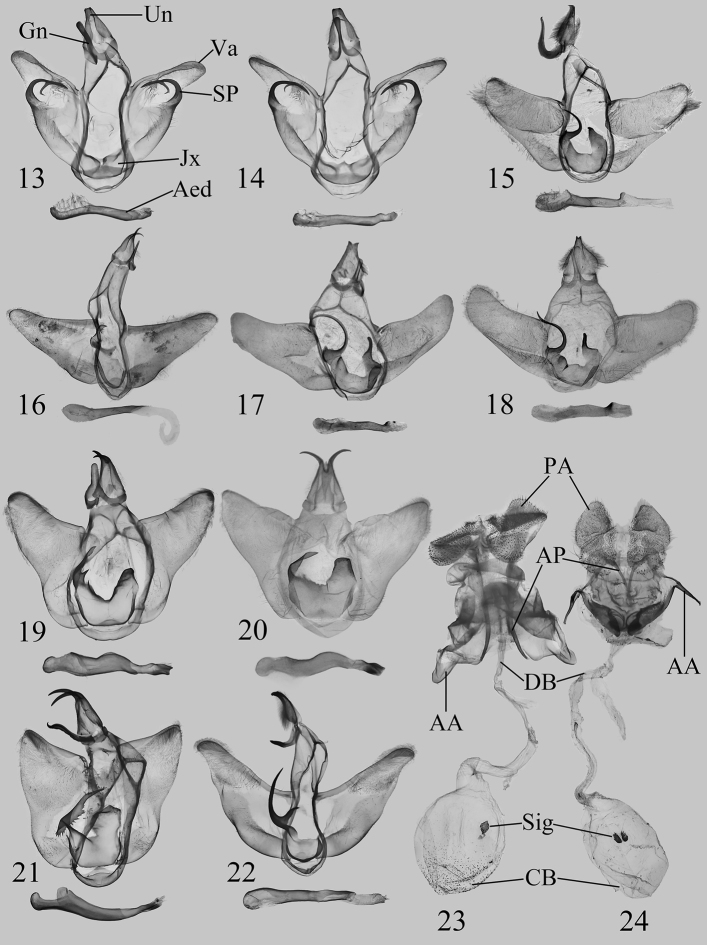
Genitalia of *Squamosa* spp.: **13, 14***S.medogensis* sp. nov., males, holotype (**13** in NEFU) and paratype (**14** in NEFU) **15***S.brevisuncayunnanensis* Wu & Fang, 2009, male, Yunnan, China, genit. prep. WuJ-090-1 (in NEFU) **16***S.ocellata* (Moore, 1879), male, Sikkim, India (in MWM/ZSM) **17***S.brevisuncabrevisunca* Wu & Fang, 2009, male, holotype (in IZCAS) **18***S.brevisuncabrevisunca* Wu & Fang, 2009 (= *S.svetlanae* Solovyev & Witt, 2009, syn. nov., male, holotype) (in MWM/ZSM) **19, 20***S.undulophallus* sp. nov., males, holotype (**19** in NEFU) and paratype (**20** in MWM/ZSM) **21***S.chalcites* Orhant, 2000, male, Chongqing, China, genit. prep. WuJ-538-1 (in NEFU) **22***S.monosa* Wu & Pan, 2015, male, Xizang, China, genit. prep. WuJ-516-1 (in NEFU) **23***S.chalcites* Orhant, 2000, female, Chongqing, China, genit. prep. WuJ-540-2 (in NEFU) **24***S.monosa* Wu & Pan, 2015, female, Xizang, China, genit. prep. WuJ-517-2 (in NEFU).

Figs 1 , 2 , 13 , 14

#### Material examined.

***Holotype*.** ♂, China, Xizang Autonomous Region, Linzhi (= Nyingchi) City, Motuo (= Medog) County, Gedang Countryside, 25.V.–5.VI.2021, leg. J. Wu and JJ. Fan, genit. prep. WuJ-519-1 (NEFU). ***Paratypes*.** 4♂, same data as for holotype, genit. prep. WuJ-520-1 (NEFU).

#### Diagnosis.

The new species can be easily distinguished from the known species by its appearance: the antennae are broadly bipectinated at basal 3/4 in male; the thorax is black mixed with a little yellow; the base and costal margin area of forewing are dark brown to black; the rounded patch located on the outside of the cell is blurry; the abdomen bears two distinct black hair tufts dorsally. In the other Asian congeners (Figs 3–12) the male antennae are bipectinated only in the basal half; the ground colour of the thorax and forewing is yellow mixed with black; the medial patch of the forewing is more distinct than in *S.medogensis* sp. nov. (Figs 1, 2); the dorsal black hair tuft on the abdomen is weak.

The male genitalia are clearly different from those of the other congeners: in *S.medogensis* sp. nov. (Figs 13, 14), the valva has a well-developed saccular process and the juxta is symmetrical. However, the same structures in other Asian species (Figs 15–22) are very different: the valvae without saccular processes; the juxta are asymmetrical with a lateral process.

#### Description.

Adult (Figs 1, 2). Wingspan 30–32 mm in male. Head yellow; labial palpus short, yellow; male antennae brown, broadly bipectinated in basal 3/4 then serrate. Thorax and tegula black mixed with a little yellow. Scales on legs black to yellow. Forewing broad, ground colour brown, wing base and costal margin area dark brown to black, outer margin area pale brown; a large, silky reddish brown, rounded medial patch located at outside of cell; subterminal line narrow, black, smoothly curved, running from costal margin near the apex to the tornus, almost parallel to outer margin, bearing a dent in the region between vines R_4_ and R_5_ towards base; wing venation visible in outer margin area, black; fringe yellow. Hindwing brown; fringe yellow. Abdomen yellow, with two distinct black dorsal hair tufts and long black hairs at terminal area.

***Male genitalia*** (Figs 13, 14). Uncus short, weakly bifid apically. Gnathos rod-shaped, blunt apically. Tegumen broad. Valva broad at base, cucullus rounded; costa slightly concave, bearing a triangular process at c. 1/3 distance from base that is covered by dense long setae; sacculus inflated, slightly sclerotised, densely covered with setae in upper half; saccular process strongly sclerotised, curved inwardly in a hook-shaped, bifid process near middle and tapering from base to apex, pointed apically. Juxta sclerotised, symmetrical, horseshoe-shaped. Saccus not obvious. Phallus slender, smoothly curved; vesica without cornuti.

***Female genitalia*.** Unknown.

#### Distribution.

**Figure 25.**
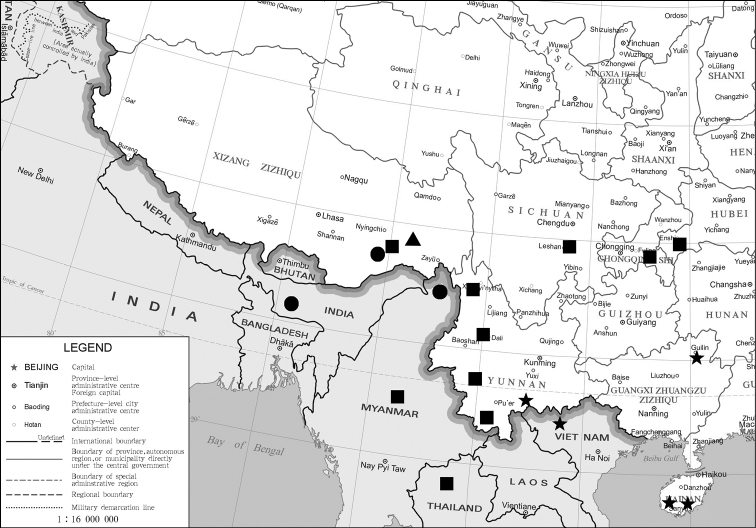
Distribution map of *Squamosa* spp.: triangle: *S.medogensis* sp. nov. (China: Xizang); circles: *S.undulophallus* sp. nov. (China: Xizang; India: Meghalaya; Myanmar: Kachin); squares: *S.chalcites* Orhant, 2000 (China: Hubei, Chongqing, Sichuan, Yunnan, Xizang; Thailand; Myanmar); stars: *S.brevisuncabrevisunca* Wu & Fang, 2009 (China: Hainan, Guangxi, Yunnan; Vietnam).

China (Xizang: Motuo) (Fig. 25).

#### Etymology.

The species is named *medogensis* for its type-locality in Motuo County, Xizang Autonomous Region, China.

#### Bionomics.

**Figures 26–28.**
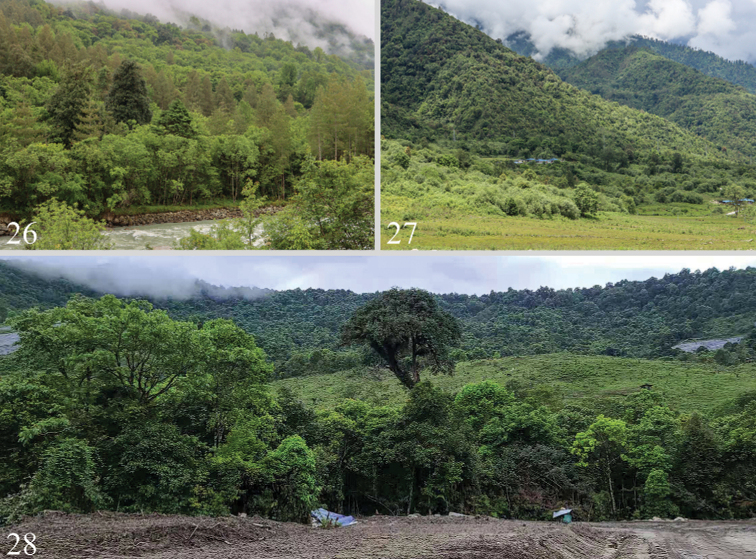
Biotopes: China, SE Xizang, Linzhi (= Nyingchi) City, Motuo (= Medog) County **26, 27** Gedang countryside, two different collecting sites of *S.medogensis* sp. nov., photograph by J. Wu **28** Beibeng Countryside, Dergong Village, biotope of *S.undulophallus* sp. nov., photograph by HL. Han.

The specimens were collected from May to June at altitudes of 1840–2120 m a.s.l., close to the subtropical evergreen broad-leaved forest, with massive shrubs, ferns and patches of grassland growing in the ground cover layer of the forest (Figs 26, 27).

#### Remarks.

According to the original descriptions, three of the diagnostic generic characters of the genus *Squamosa* are: the antennae bipectinated only at the basal half in males, the valva without saccular process, and the juxta with lateral asymmetrical processes (Solovyev and Witt 2009; Wu and Fang 2009). *Squamosamedogensis* sp. nov. does not match any of the above three characters, but since all other typical characters for this genus were observed, we therefore tentatively place this new species in *Squamosa*.

### ﻿ Squamosa undulophallus sp. nov.

Taxon classificationAnimaliaLepidopteraLimacodidae

764B2FDB-7122-5524-B2DD-7FF46F93F48F

http://zoobank.org/04666165-960B-4AFA-BE73-DFBB9F61C6C9

Figs 7 , 8 , 19 , 20

#### Material examined.

***Holotype*.** ♂, China, Xizang Autonomous Region, Linzhi (= Nyingchi) City, Motuo (= Medog) County, Beibeng Countryside, Dergong Village, 850 m a.s.l., 25.V.–4.VI.2021, leg. HL. Han, genit. prep. WuJ-518-1 (NEFU). ***Paratypes*.** 1♂, India, sept. or. W. Meghalaya, Garo Hills, Nokrek Nat. Park, 25°40'N, 91°04'E, 2–13.VII.1997, 1150 m a.s.l., leg. Afonin and Siniaev, genit. prep. 16149 (MWM/ZSM); 1♂, Myanmar, Putao, 550 m a.s.l., 27. IV. 1998, leg. Murzin and Sinjaev, genit. prep. 16169 (MWM/ZSM); 1♂, Myanmar, 16 km E Putao, 500 m a.s.l., 28–30.IV.1998, leg. Murzin and Sinjaev, genit. prep. 16230 (MWM/ZSM); 1♂, Myanmar, 21 km E Putao, Nan Sa Bon Village, 550 m a.s.l., 1–5.V.1998, leg. Murzin and Sinjaev, genit. prep. 16150 (MWM/ZSM); 1♂, Myanmar, 25 km E Putao, env. Nan Sa Bon Village, 800 m a.s.l., 6–9. V. 1998, leg. Murzin and Sinjaev, genit. prep. 16231 (MWM/ZSM).

#### Diagnosis.

The new species is very similar to *S.chalcites* (Figs 9, 12) in appearance, but it can be distinguished from the latter by the following characters: middle of costal margin area of forewing is covered by dense black scales and the medial patch of forewing is conspicuous. In *S.chalcites*, the forewing only bears small scattered black scales and the medial patch of forewing is blurry.

#### Description.

Adult (Figs 7, 8). Wingspan 35–39 mm in male. Head brown; labial palpus short, brown; male antennae brown, bipectinated at basal half then serrate. Thorax dark brown to black dorsally, mesothorax with conspicuous tuft of long black hairs anteriorly; tegula brown. Scales on legs dark brown to pale yellow. Forewing distinct elongate, ground colour dark brown mixed with numerous black scales, especially dense in middle part of costal margin area, outer margin area pale brown; a conspicuous, large, silky, rounded medial patch located at outside of cell, inner half bluish black, outer half reddish brown with an arched bright line embedded in middle; subterminal line narrow, black, with depressions in the region of vein R_4,_ and slightly concave between veins M_3_ and CuP; two distinct black spots at middle of veins CuP and 1A+2A; fringe brown. Hindwing ground colour greyish brown to dark brown, anal margin area is darker; fringe pale brown. Abdomen brown to dark brown, mixed with little black hairs dorsally and long black hairs at terminal area.

***Male genitalia*** (Figs 19, 20). Uncus short, with apex deeply bifid, strongly sclerotised. Gnathos finger-shaped or slightly widened near apex, blunt apically. Tegumen broad. Valva short and broad; sacculus swollen at base, without saccular process; costa slightly concave at middle; cucullus narrow and rounded. Juxta asymmetrical, horseshoe-shaped, central depression V-shaped; left process strongly sclerotised, long plate-shaped, bearing a distinct spur near base in some individuals; right process plate-shaped, strongly sclerotised apically. Vinculum narrow. Saccus not obvious. Phallus thick, conspicuously waved, slightly thinner near apex, strongly sclerotised and somewhat bifid terminally.

***Female genitalia*.** Unknown.

#### Distribution.

China (Xizang: Motuo), India (Meghalaya), Myanmar (Kachin) (Fig. 25).

#### Etymology.

The species is named *undulophallus* after its distinctly wavy phallus.

#### Bionomics.

The specimens were collected from April to July at altitudes about 550–1150 m a.s.l. The collection area in China is a subtropical climate zone (Fig. 28).

### ﻿ Squamosa chalcites

Taxon classificationAnimaliaLepidopteraLimacodidae

Orhant, 2000

704BB84D-33C1-53E8-A6F4-16F904E0E5E2

Figs 9 , 12 , 21 , 23

Squamosa chalcites Orhant, 2000. Lambillionea (100) 3: 471. Type locality Myanmar: Maymyo.

#### Specimens examined.

1♂, China, Prov. Yunnan, Pu’er City, Manxieba Village, 3.VI.2018, leg. HL. Han, J. Wu and MR. Li, genit. prep. WuJ-109-1 (NEFU); 1♂, China, Chongqing Municipality, Mt. Simian, 23.VII–6.VIII.2018, leg. GX. Wang and WJ. Li, genit. prep. WuJ-539-1 (NEFU); 2♂, China, Chongqing Municipality, Mt. Simian, 24–30.VII.2019, leg. TT. Zhao and SC. Deng, genit. prep. WuJ-538-1 (NEFU); 4♀, China, Chongqing Municipality, Mt. Simian, 29.VII.–2.VIII.2020, leg. HL. Han and J. Wu, genit. prep. WuJ-540-2 and 541-2 (NEFU).

***Female genitalia*** (Fig. 23). Papillae anales flattened, foot-shaped, covered with dense hairs on surface. Postvaginal plate flattened, strongly sclerotised. Apophysis anterioris highly modified, short, tongue-shaped; apophysis posterioris long and slender, c. 3 × length of apophysis anterioris. Ductus bursae long, membranous, not spiral-shaped. Corpus bursae pear-shaped, twisted in its apical part, with a strongly sclerotised, nearly elliptical central signum.

#### Distribution.

China (Hubei, Chongqing, Sichuan, Yunnan, Xizang); Thailand, Myanmar (Fig. 25).

#### Remarks.

Although the female adult was described in Orhant (2000), the female genitalia are described herein for the first time. In contrast to another known female of the genus, *S.monosa*, two distinctive features of this species can be recognised: the apophysis anterioris is highly modified, tongue-shaped; and the corpus bursae only with a single signum. However, in the female genitalia of *S.monosa* (Fig. 24), the apophysis anterioris is slender and the corpus bursae has a pair of signa.

### ﻿ Squamosa brevisunca

Taxon classificationAnimaliaLepidopteraLimacodidae

Wu & Fang, 2009

888A6FFC-00AB-5BE1-A614-EAFD233764D6

Figs 4 , 5 , 6 , 15 , 17 , 18

Squamosa brevisunca Wu & Fang, 2009, Acta Zootaxonimica Sinica 34 (2): 237. Type locality China: Hainan.
Squamosa ocellata (not Moore): Cai 1981: 99, fig. 648.

### ﻿ Squamosa brevisunca brevisunca

Taxon classificationAnimaliaLepidopteraLimacodidae

Wu & Fang, 2009

433341A9-E878-5D64-B97F-48A3AFF1B9D5

Figs 4 , 5 , 17 , 18

Squamosa brevisunca brevisunca Wu & Fang, 2009, Acta Zootaxonimica Sinica 34 (2): 237. Type locality China: Hainan. Holotype (by original designation): ♂ (IZCAS) [examined].
= Squamosasvetlanae Solovyev & Witt, 2009, syn. nov., Entomofauna, suppl. 16: 186. Type locality Nord-Vietnam: Mt. Fan-si-pan. Holotype (by original designation): ♂ (MWM/ZSM) [examined].

#### Specimen examined.

1♂, China, Prov. Yunnan, Lvchun County, Mt. Huanglian, 27–31.VII.2018, leg. HL. Han, J. Wu, MR. Li, genit. prep. WuJ-091-1 (NEFU).

#### Diagnosis.

The nominate subspecies cannot be distinguished from *S.brevisuncayunnanensis* Wu & Fang, 2009 (Fig. 6) externally but the morphology of male genitalia is diagnostic. In the male genitalia, the left process of juxta is long and the right process is finger-shaped apically in *S.brevisuncabrevisunca* (Figs 17, 18), whereas the left process of juxta is short and the right process is blunt apically, without the finger-shaped apex in *S.brevisuncayunnanensis* (Fig. 15).

#### Distribution.

China (Hainan, Guangxi, Yunnan), Vietnam (Fig. 25).

#### Bionomics.

We collected a single specimen in July at altitude about 1945 m a.s.l., with a light trap close to a broad-leaved forest with ferns and shrubs.

#### Remarks.

By examining the holotypes of *S.brevisuncabrevisunca* (China: Hainan) and *S.svetlanae* (Vietnam: Mt. Fan-si-pan), we found that there are no significant differences in either the external appearance or the morphology of the male genitalia between them. In addition, we also collected a male specimen of the nominate subspecies from southern Yunnan, China, an area extremely close to the type locality of *S.svetlanae*, on the basis of which we establish the synonymy *S.brevisuncabrevisunca* Wu & Fang, 2009 = *S.svetlanae* Solovyev & Witt, 2009 syn. nov. here.

### ﻿Key to the Asian species of *Squamosa* based on male genitalia, with distributions

**Table:** 

1	Valva without saccular process, juxta asymmetrical	**2**
–	Valva with saccular process, juxta symmetrical	***S.medogensis* sp. nov.** (China: Xizang)
2	Uncus slightly bifid	**3**
–	Uncus deeply bifid	**4**
3	Juxta with both lateral processes	**5**
–	Juxta with a single left lateral process	***S.monosa* Wu & Pan** (China: Xizang)
4	Valva short and broad; tegumen short and broad; phallus sinuous	**6**
–	Valva elongate; tegumen long and narrow; phallus slender	***S.ocellata* (Moore)** (India, Nepal, Bhutan, Myanmar)
5	Left process of juxta long; right process finger-shaped apically	***S.brevisuncabrevisunca* Wu & Fang** (China: Hainan, Guangxi, Yunnan; Vietnam)
–	Left process of juxta short; right process blunt apically, without finger-shaped apex	***S.brevisuncayunnanensis* Wu & Fang** (China: Yunnan)
6	Left process of juxta sawblade-shaped; phallus smoothly curved	***S.chalcites* Orhant** (China: Hubei, Chongqing, Sichuan, Yunnan, Xizang; Thailand, Myanmar)
–	Left process of juxta long plate-shaped; phallus distinctly waved	***S.undulophallus* sp. nov.** (China: Xizang, India: Meghalaya, Myanmar: Kachin)

## Supplementary Material

XML Treatment for Squamosa

XML Treatment for Squamosa medogensis

XML Treatment for Squamosa undulophallus

XML Treatment for Squamosa chalcites

XML Treatment for Squamosa brevisunca

XML Treatment for Squamosa brevisunca brevisunca
